# A phase I clinical trial of continual alternating etoposide and topotecan in refractory solid tumours

**DOI:** 10.1038/sj.bjc.6602671

**Published:** 2005-06-28

**Authors:** R T Penson, M V Seiden, U A Matulonis, L J Appleman, A F Fuller, A Goodman, S M Campos, J W Clark, M Roche, J P Eder

**Affiliations:** 1 Division of Hematology and Oncology, Department of Medicine, Massachusetts General Hospital, Boston, MA 02114, USA; 2 Brigham and Women's Hospital Division of Gynecologic Oncology, Boston, MA 02114, USA; 3 Dana-Farber Cancer Institute and Division of Adult Oncology, Department of Medicine and the Dana Farber/Harvard Cancer Center, Boston, MA 02114, USA; 4 Department of Gynecology and Obstetrics, Massachusetts General Hospital, Boston, MA 02114, USA

**Keywords:** sequential, palliative, chemotherapy, rational, topoisomerase

## Abstract

The goal of this phase I study was to develop a novel schedule using oral etoposide and infusional topotecan as a continually alternating schedule with potentially optimal reciprocal induction of the nontarget topoisomerase. The initial etoposide dose was 15 mg m^−2^ b.i.d. days (D)1–5 weeks 1,3,5,7,9 and 11, escalated 5 mg per dose per dose level (DL). Topotecan in weeks 2,4,6,8,10 and 12 was administered by 96 h infusion at an initial dose of 0.2 mg m^−2^ day^−1^ with a dose escalation of 0.1, then at 0.05 mg m^−2^ day^−1^. Eligibility criteria required no organ dysfunction. Two dose reductions or delays were allowed. A total of 36 patients with a median age of 57 (22–78) years, received a median 8 (2–19) weeks of chemotherapy. At DL 6, dose-limiting toxicities consisted of grade 3 nausea, vomiting and intolerable fatigue. Three patients developed a line-related thrombosis or infection and one subsequently developed AML. There was no febrile neutropenia. There were six radiologically confirmed responses (18%) and 56% of patients demonstrated a response or stable disease, typically with only modest toxicity. Oral etoposide 35 mg m^−2^ b.i.d. D1–5 and 1.8 mg m^−2^ 96 h (total dose) infusional topotecan D8–11 can be administered on an alternating continual weekly schedule for at least 12 weeks, with promising clinical activity.

Etoposide and topotecan are both schedule-dependent cytotoxics that have a broad spectrum of antineoplastic activity. Both interact with topoisomerases, essential nuclear enzymes that cleave DNA to reduce torsional strain during replication and recombination. Topotecan interacts with topoisomerase I (topo I), inducing reversible single-stranded breaks and a compensatory upregulation in topoisomerase II (topo II). (Liu, 1989) Etoposide binds to topo II to cause double-stranded DNA breaks with a compensatory increase in topo I. This see-saw compensation occurs with a delay such that sequential treatment may offer the optimal schedule of treatment. (Eder *et al*, 1998) Continual exposure to schedule-dependent cytotoxics was first shown to be effective for acute lymphocytic leukaemia of childhood and since then, the investigation of protracted exposure to such agents has been the centre of much of the investigation of the pharmacodynamics of chemotherapy. (Furman *et al*, 1976; Collins *et al*, 1990). With the positive experience of sequencing the two topoisomerase interacting agents, doxorubicin and topotecan (Seiden *et al*, 2002), this study was conceived, building on the anticipation that continual exposure may maximise the benefit of repeatedly alternating topoisomerase I and II interacting drugs, and to define the maximum-tolerated dose (MTD).

DNA topoisomerases are nuclear enzymes essential for DNA replication, RNA transcription, chromosomal condensation and mitotic chromatid separation (Caron and Wang, 1993; Gupta *et al*, 1995; Kohn and Pommier, 2000). There are two major classes of topoisomerases, I and II, in eukaryotic cells. Topoisomerase I relaxes DNA by forming a covalent bond with the 3′-terminus of a DNA nucleotide, producing a single-stranded break (Gupta *et al*, 1995). Topoisomerase II functions as a dimer and forms a double-stranded cleavage with each topoisomerase II molecule covalently bound to the 5′-terminus of a DNA nucleotide, producing a double-stranded break (Holm *et al*, 1985). Topoisomerase-targeted agents stabilise a transient covalent enzyme–DNA complex, which produces DNA strand cleavage and apoptosis (Kohn and Pommier, 2000). Preclinical study of the *in vivo* therapeutic effect of sequential combinations of topoisomerase I- and II-acting drugs in a murine tumour model system reveals that topoisomerase I mRNA and protein levels in the tumour decrease, whereas topoisomerase II mRNA and protein levels rise, after treatment with camptothecins (Eder *et al*, 1998) and topotecan (Liebes *et al*, 1998). The reverse effect, a fall in topoisomerase II and concurrent rise in topoisomerase I levels with a topoisomerase II-active drug treatment, is observed, with the suggestion that sequentially combined topoisomerase I and II agents may result in greater than additive tumour cytotoxicity with relatively little increase in toxicity. *In vitro* studies suggest topoisomerase II*α* is cell cycle phase dependent, being only present in the S phase and G_2_–M phases of the cell cycle in contrast to topoisomerases I and II*β*, which are expressed constitutively throughout the cell cycle (Heck and Earnshaw, 1986; Liu, 1989). Agents interacting with any topoisomerase exhibit cell cycle cytotoxic specificity. However, there is considerable variation, dependent on dose and schedule, with notable differences between i*n vitro* and *in vivo* observations (Kaufmann, 1991).

The first clinical trials combining topoisomerase I and II inhibitors have shown substantial, albeit nonlethal, toxicity (Ando *et al*, 1997). These studies have focused on etoposide as the topoisomerase II active agent and have used the camptothecin analogue first. Sequential treatment appeared to have synergistic effects with a suggestion of greater than additive tumour cytotoxicity and no increase in host toxicity. In Seiden *et al*'s study, the alternative combination of doxorubicin and topotecan was chosen because of *in vitro* observations that firstly, a sequence of doxorubicin (4 days) followed by camptothecin (4 days) produced the greatest tumour growth delay in a murine breast cancer cell line (>50%) with no increase in toxicity and secondly, changes in topoisomerase I levels recovered more rapidly than topoisomerase II levels (Eder *et al*, 1998; Seiden *et al*, 2002).

A phase I trial of sequentially administered etoposide and topotecan was therefore constructed on these observations. The objectives of the study were to determine the dose-limiting toxicities (DLTs) and MTD of sequential etoposide (days (D)1–5) and topotecan by 96 h CIV (D8–11) q 14 in patients with refractory solid tumour malignancies.

## PATIENTS AND METHODS

### Eligibility criteria

The study protocol was reviewed and approved by the Dana Farber/Partners CancerCare (Boston, MA, USA) Institutional Review Board. An informed consent document satisfying all federal and institutional requirements was read by the patients and signed as a condition of their registration. Patients had histologically documented metastatic or inoperable malignant solid tumours, for which there was no known curative or standard palliative therapy. Performance status was Eastern Cooperative Oncology Group 0–2. Patients had adequate hepatic, renal and haematological function determined by a serum glutamate-oxalo-acetate transaminase <2.5 × upper limit of normal, bilirubin <1.5 × upper limit of normal, creatinine clearance >50 ml min^−1^, an ANC >1500 *μ*l^−1^ and platelets >150 000 *μ*l^−1^. Patients had to be more than 3 weeks from last chemotherapy, 2 weeks from surgery, 6 weeks from nitrosoureas or radiotherapy and could not have had prior pelvic radiotherapy. Women of childbearing potential could not be pregnant or lactating and fertile participants had to practise adequate contraception. Additional inclusion criteria included age >18 years and having central venous access; patients had to be capable of taking oral medication with an anticipated life expectancy in excess of 2 months. Eligibility tests had to be performed within 21 days of commencing therapy.

### Treatment plan

**Figure 1:**
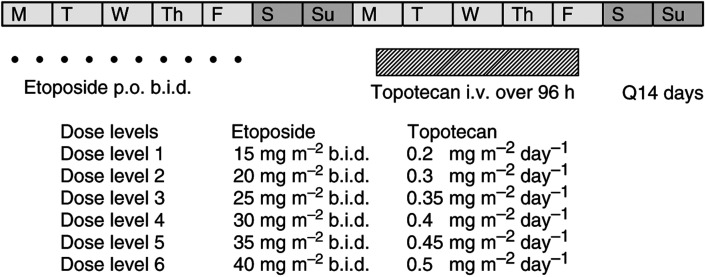
Treatment schema. Legend: M, Monday; T, Tuesday; W, Wednesday; Th, Thursday; F, Friday; S, Saturday and S, Sunday. Etoposide administered orally as the i.v. preparation.

The treatment schema is illustrated in Figure 1. Patients had a history, physical examination, complete blood count and serum chemistries (including liver function tests, Mg^2+^ and creatinine) performed before each week of therapy, and complete blood count with differential was performed twice each week. These assessments were repeated at 1 month after the last course of treatment and on apparent progression. Documentation of all measurable disease by examination and any appropriate imaging studies (e.g. plain radiograph, computerised tomography and nuclear medicine scan) and EKG were performed before therapy, at week 7 and 13.

On weeks 1, 3, 5, 7, 9 and 11, etoposide was administered orally per allocated dose level (DL) as 10 doses in 5 days with b.i.d. dosing using the i.v. preparation in sterile prefilled syringes. The i.v. preparation of etoposide was used instead of the 50 mg softgel capsules (VePesid®), to allow the necessary dose increments required for the study. Patients were instructed to squirt the contents of a syringe into orange juice and drink it, and store the etoposide at room temperature. On weeks 2, 4, 6, 8, 10 and 12, topotecan was administered by 96-h i.v. infusion as per allocated DL. The 96-h i.v. infusion was chosen to allow ambulatory administration of chemotherapy rather than attending for the standard D1–5 30-min infusions of chemotherapy. Growth factors were not allowed to maintain dose intensity. Etoposide (VePesid™; Bristol-Myers Squibb, Princeton, NJ, USA) and topotecan (Hycamptin™; Smith Kline Beecham, King of Prussia, PA, USA) were prepared from commercially available supplies, formulated and administered per institutional guidelines. Antiemetic therapy could include prn lorazepam 0.5–2.0 mg i.v. and/or prochlorperazine 10 mg p.o. or perphenazine 4 mg p.o. as per institutional standards. The administration of glucocorticoids or 5HT_3_ antagonists as antiemetics was permitted only after the failure of other antiemetic agents. All patients were treated with warfarin 1 mg p.o. as prophylaxis against line-related thrombosis unless fully anticoagulated for another indication.

Initial DL was 15 mg m^−2^ b.i.d. etoposide D1–5 with topotecan D8–12 at 0.2 mg m^−2^ day^−1^ q 14. Etoposide was then increased in subsequent DLs in increments of 5 mg m^−2^ dose^−1^ and topotecan was increased to 0.3 mg m^−2^ day^−1^ for DL 2 and then in 0.05 mg m^−2^ day^−1^ increments. Initially, three patients fully evaluable were to be recruited to DL 1. If there was no DLT, then accrual continued until the MTD was defined. Dose-limiting toxicities were defined as Gr III neutropenia for >72 h, Gr III thrombocytopenia and Gr III nonhaematological toxicity by CTC 2.0 criteria. Subsequent cohorts of patients were accrued if DLT was not observed in preceding patients over the first 4 weeks of treatment. If DLT was observed, two further patients were enrolled to that DL and the occurrence of a second DLT in 2–6 patients established the previous dose as the MTD. In total, 10 additional patients were treated at the MTD to better define the profile of toxicity at this dose.

Dose modifications were not allowed during the first four weeks of therapy, during which time DLT was evaluated. After week 4, dose modifications were allowed and based on the neutrophil and platelet counts on the first and the fourth day of each treatment week. If the ANC was <1000 *μ*l^−1^ or platelet count was <100 000 *μ*l^−1^ on D1, no treatment was given that week and a 25% dose reduction was required for either drug if the ANC was 1000–1499 *μ*l^−1^ or platelet count was 100 000–149 999 *μ*l^−1^. Treatment was stopped on D4 of each week of treatment if the ANC was <1499 *μ*l^−1^ or platelet count was <149 999 *μ*l^−1^.

Therefore, as the day 4 counts were available after 7/10 doses of etoposide and approximately 75/96 h of topotecan, discontinuation of treatment on day 4 constituted a total dose reduction for that cycle of approximately 44%. Patients requiring more than one dose reduction for a given drug were removed from the study. For the 10 additional patients treated at the MTD, dose reductions were allowed during the first 4 weeks of treatment at the discretion of the treating physician. There were no planned dose escalations.

### Disease response criteria

For disease measurable radiologically or by examination, response to therapy was defined in the following manner by standard WHO criteria. A complete response required the disappearance of all measurable disease. For patients with ovarian cancer, a serum CA-125 concentration <35 U l^−1^ for a minimum of 30 days was also required. Partial response was a reduction in tumour burden of 50% or greater for a minimum of 30 days and decrease in serum CA-125 by at least 50% for patients with an elevated marker. Progressive disease was indicated by a greater than 25% increase in tumour burden or serum tumour marker or the appearance of any new lesion. Stable disease was defined as a decrease in tumour burden or serum tumour marker level not meeting partial response criteria or an increase that did not constitute progressive disease. The following definitions were employed in situations where disease could not be measured by radiological techniques. A complete response required normalisation of tumour marker and complete resolution of evaluable disease such as pleural fluid or ascites, if present. Partial response was a greater than 50% decrease in serum tumour marker, with a reduction in ascites or pleural effusion. A confirmed rising serum tumour marker, even without confirmation by radiological or physical examination, was considered progressive disease.

## RESULTS

Between October, 1999 and August, 2002, 36 patients were enrolled. A total of 287 weeks of chemotherapy were delivered (median 8 (2–19) weeks).

### Patient characteristics

The characteristics of the 36 patients enrolled into the study are summarised in Table 1. The median age was 57 years (range, 22–78). All patients were evaluable for toxicity. In all, 27 patients had ovarian cancer, four had sarcoma, three had non-small-cell lung cancer and one each had thymoma and cholangiocarcinoma. All patients had progression of tumour with at least one prior chemotherapy regimen, and 16 patients had received more than two prior chemotherapy regimens. Only two patients had ECOG performance score 2.

Additional patients were enrolled to DLs 1 and 2 to replace patients who developed rapid disease progression. One patient developed small bowel obstruction at week 3, one patient developed symptomatic progression of brain metastases at week 3. At DL 4, the study seemed to be near defining the MTD with haematologic toxicity-triggered dose reductions. However, these prevented significant toxicity and the protocol was revised to mandate no dose reductions during the first 4 weeks of therapy. Despite this, DL 4 appeared tolerable and the dose was escalated after a total of nine patients had been enrolled at this DL.

### Toxicity

Haematologic and nonhaematologic toxicity are recorded in Tables 1, 2 and 3. Grade 4 haematologic toxicity did not occur, despite continual chemotherapy, effectively prevented by the dose delay and reduction rules. No patient required hospitalisation or antibiotics for neutropenia and there was no febrile neutropenia. Significant nonhaematological toxicity was relatively infrequent. Mild alopecia was common. Six patients experienced mild (one grade 2) infusion catheter-related discomfort or swelling. One patient had a problematic paraneoplastic plexopathy, making evaluation of line-related symptoms difficult, and two patients developed catheter-related infection, both managed conservatively with outpatient oral antibiotic therapy with preservation of the line. Only one catheter-related thrombosis was documented. Although mucositis was notably rare, an unpleasant metallic taste was prominent during treatment with etoposide. At DL 6, continuous nausea, vomiting and fatigue were intolerable. One patient with primary peritoneal cancer and a past history of endometrial cancer subsequently developed AML with normal cytogenetics.

### Tumour response

A total of 34 patients were evaluable for response (Table 4). Two were inevaluable because of the interval development of small bowel obstruction and cerebral metastases, after 2 and 3 weeks of therapy, respectively. Overall, six of 34 patients' (18%) tumours demonstrated a response, all but one of which were confirmed 1 month later on imaging, and one of which was a complete response. The duration of response was a median 8.1 months (range 6.8–21.5 months). Tumours in many patients developed minor responses and a response or stable disease was observed in 56% of patients. All of the tumour responses were observed in patients with ovarian cancer, for an overall response rate of 23% in this subgroup. Of the evaluable patients with ovarian cancer, nine (35%) had been treated with three or more prior lines of therapy.

## DISCUSSION

The recent availability of an increasing number of promising investigational and newly approved drugs has rechallenged the role of multiagent chemotherapy in the clinical management of solid tumours with greater impetus for rational combinations of chemotherapy that maximise efficacy with minimal toxicity (Cannistra, 2002). However, the best approach for combining topotecan with other anticancer agents has been debated because additive myelosuppresssion can markedly limit the doses of drugs that can be safely administered together (Cannistra, 1999). Etoposide and topotecan are both commonly used agents with potential synergy. This study demonstrates that oral etoposide 35 mg m^−2^ b.i.d. D1–5 and 1.8 mg m^−2^ 96 h (total dose) infusional topotecan can be administered on an alternating continual weekly schedule for at least 12 weeks, with promising clinical activity. Furthermore, this potentially maximises the benefit of alternating induction of topoisomerase I and II. The regimen was found to be a well tolerated, although a relatively inconvenient, outpatient treatment. Toxicity is characterised by neutropenia, and thrombocytopenia was dose-limiting with nonhaematological toxicity being notably mild.

DNA topoisomerases I and IIa are targets for many clinically important antineoplastic agents. The drugs that interact with topoisomerase I, the camptothecins, are structurally distinct from the topoisomerase II-interacting agents of the anthracycline, epipodophyllotoxin and anthracenedione classes. No clinically useful agent that preferentially targets topoisomerase II*β* has been developed to date. Maximising the utility of topoisomerase-interacting agents, by sequential combination, may also minimise the development of resistance (Potmesil *et al*, 1988; Sugimoto *et al*, 1990). Although p-glycoprotein overexpression is likely the most important, other mechanisms probably also contribute, and continual, rather than intermittent exposure may limit the development of resistance (Schneider *et al*, 1994). Continual exposure will also limit the development of resistance. However, whether significant benefit comes from a greater total dose, longer exposure of a greater number of cycling cells or time above a concentration threshold is unclear (Joel, 1996).

The rationale for choice of DLs appears to have been justified by the results. Protracted oral etoposide dose in combination treatment is typically dosed at 50–75 mg m^−2^ day^−1^ (Hainsworth, 1999). There is limited data on topotecan by protracted infusion in combination regimens. Hochster *et al* reported an MTD of 0.4 mg m^−2^ day^−1^ D1–14 q 21 in combination with short infusion paclitaxel (Chachoua *et al*, 1997) and we defined an MTD for topotecan of 0.7 mg m^−2^ day^−1^ by 96 h infusion in combination with 96 h infusion paclitaxel (Penson *et al*, 2001). Doxorubicin and topotecan administered sequentially could be combined at 60–70% of the typical dose (Seiden *et al*, 2002). With the protocol design calling for protracted sequential treatment without a pause to allow for recovery of bone marrow function, the starting doses were reduced a further 50%. The dose reduction strategy set stringent thresholds for continued treatment in an attempt to prevent severe myelosuppression. Given the variable and unpredictable bioavailability of oral etoposide, and the resulting variable tolerability, there was surprisingly little intrapatient variability in haematological and nonhaematological toxicity during the study. However, on three occasions, the DL cohorts had to be modified to accrue more patients.

Topotecan appears to be a schedule-dependent cytotoxic (Rowinsky *et al*, 1992). Phase I and II clinical trials have been performed to evaluate the administration of topotecan by continuous i.v. infusion for periods of 24 h (van Warmerdam *et al*, 1995; Abbruzzese *et al*, 1996; Hoskins *et al*, 1998), 72 h (Burris *et al*, 1994), 120 h (Burris *et al*, 1994) and 21 days (Hochster *et al*, 1994). The Canadian National Cancer Institute conducted a randomised phase II trial to investigate the schedule dependence of topotecan (Hoskins *et al*, 1998). Patients received topotecan given according to the approved dosing regimen, 1.5 mg m^−2^ i.v. over 30 min on 5 consecutive days every 21 days, or by 24-h i.v. infusion of a 1.75 mg m^−2^ dose once a week for 4 weeks, with cycles repeated every 6 weeks. The results revealed that the response rate for topotecan given by the D1–5 dosing regimen was significantly better than the 24-h infusion schedule (22.6 *vs* 3.1%, *P*=0.026). The weekly 24-h infusion schedule was clearly associated with less severe haematological toxicity than the approved dosing regimen. However, the investigators did not believe that the dose, which had been previously defined in a phase I study (Haas *et al*, 1994), could be significantly increased. In contrast, there are more encouraging results from studies of topotecan delivered by protracted infusion, in combination with other chemotherapeutic agents (Hochster *et al*, 1994), and our own experience with 96 h infusion topotecan has been very positive (Penson *et al*, 2001).

While the hope has been to exploit a therapeutic window, combining topoisomerase I and II inhibitors has been associated with substantial toxicity. Studies have, in fact, not infrequently defined an MTD lower than the initial DL (Karato *et al*, 1993; Ando *et al*, 1997). The commonest combinations have been irinotecan given concurrently with etoposide (Karato *et al*, 1993; Masuda *et al*, 1994; Oshita *et al*, 1997; Masuda *et al*, 1998), in which the DLTs were typically neutropenia and diarrhoea, and required G-CSF support. A study of sequential irinotecan followed by etoposide reported a similar MTD (Ando *et al*, 1997). Other sequential studies have investigated topotecan and etoposide (Herben *et al*, 1997; Hammond *et al*, 1998) or doxorubicin and topotecan (Tolcher *et al*, 1997; Seiden *et al*, 2002). This study reports a particularly favourable toxicity profile, with encouraging efficacy. Although oral etoposide potentially offers a superior schedule of dosing (Slevin *et al*, 1989), this has been questioned (Girling, 1996). Limited data is available about oral topotecan. However, oral topotecan, or an alternate bioavailable camptothecin analogue, would obviate the logistical challenges of infusional chemotherapy and this option is an obvious avenue of investigation and is being discussed.

In a heavily pretreated population of patients, the response rate was impressive. There were six radiologically confirmed responses (18%), three of which were confirmed complete radiological remissions. The majority (56%) of patients had a tumour response or stable disease, typically tolerated with minimally toxic treatment. Although little more than a proof of principle, the approach of delivering minimally toxic treatment to ‘hold the disease at bay’, or ‘shrink’ the disease may have delivered blood concentrations above the therapeutic threshold but below those associated with significant toxicity. However, this therapeutic window is far from defined, and is complex given the attempt to concurrently escalate both drugs.

In summary, this study has demonstrated that oral etoposide 35 mg m^−2^ b.i.d. D1–5 and 1.8 mg m^−2^ 96 h (total dose) infusional topotecan can be administered on an alternating continual weekly schedule for at least 12 weeks with promising clinical activity that merits a phase II study in patients with ovarian cancer.

## Figures and Tables

**Table 1:** Patient characteristics

**Characteristic**	**Value**
*Patients*	
*n*=	36
	
*Age (years)*	
Median	54
Range	42–70
	
*Performance status (n)*	
0	14
1	19
2	2
Unknown	1
	
*Tumour site (n)*	
Ovarian cancer	27 (75%)
Sarcoma	4 (11%)
Non-small-cell lung cancer	3 (8%)
Thymoma	1 (3%)
Cholangiocarcinoma	1 (3%)
	
*Prior chemotherapy regimens (n)*	
Median	2
Range	1–6

**Table 2:** Frequency of worst grade of haematological toxicity

	**Doses**			**# occurrences of toxicity (grade 1/2/3/4)**		
**DL**	**Etoposide (mg m^−2^ dose^−1^)**	**Topotecan (mg m^−2^ day^−1^)**	**# patients**	**Anaemia**	**Neutropenia**	**Thrombocytopenia**
1	15	0.2	4	2/2/0/0	1/1/0/0	0/0/0/0
2	20	0.3	4	0/1/0/0	1/0/0/0	0/0/0/0
3	25	0.35	3	1/2/0/0	0/2/1/0	2/0/0/0
4	30	0.4	9	2/4/1/0	3/3/0/0	1/1/0/0
5	35	0.45	3	1/1/0/0	0/1/0/0	0/1/0/0
6	40	0.5	3	1/3/0/0	0/1/1/0	1/0/0/0
						
MTD	35	0.45	10	3/6/1/0	1/3/4/0	1/2/0/0

DL=dose level; MTD=maximum-tolerated dose.

**Table 3:** Frequency of worst grade of non-haematological toxicity

	**# occurrences of toxicity (grade 1/2/3/4)**					
**DL**	**# patients**	**Fatigue**	**Nausea**	**Vomiting**	**Diarrhea**	**Mucositis**
1	4	1/2/0/0	1/1/0/0	0/1/1/0	0/2/0/0	0/0/0/0
2	4	3/1/0/0	3/0/0/0	2/0/0/0	0/0/0/0	0/0/0/0
3	3	1/2/0/0	1/2/0/0	2/1/0/0	2/1/0/0	1/0/0/0
4	9	2/4/2/0	3/6/0/0	1/3/0/0	3/3/1/0	0/0/0/0
5	3	2/1/0/0	2/1/0/0	1/0/0/0	0/2/0/0	0/0/0/0
6	3	1/3/0/0	2/1/1/0	1/1/1/0	1/1/0/0	1/0/0/0
MTD	10	2/5/3/0	5/4/1/0	3/1/0/0	1/1/0/0	2/0/0/0

DL=dose level; MTD=maximum-tolerated dose.

**Table 4:** Response to therapy

**Characteristic**	**Value (%)**
*Outcome*	
Complete response	1 (3%)
Partial response	5 (15%)
Stable disease	14 (41%)
Progressive disease	15 (44%)
Not evaluable	2 (6%)
Overall response rate % (95% CI)	18 (6–31%)
